# Nicotinic Receptors: Role in Addiction and Other Disorders of the Brain

**Published:** 2008-11-11

**Authors:** Geeta Sharma, Sukumar Vijayaraghavan

**Affiliations:** Department of Physiology and Biophysics and the Neuroscience Program, University of Colorado, Denver, School of Medicine Aurora CO 80045.

**Keywords:** Nicotine, drug abuse, network, hedonic homeostasis, Calcium

## Abstract

Nicotine, the addictive component of cigarette smoke has profound effects on the brain. Activation of its receptors by nicotine has complex consequences for network activity throughout the brain, potentially contributing to the addictive property of the drug. Nicotinic receptors have been implicated in psychiatric illnesses like schizophrenia and are also neuroprotective, potentially beneficial for neurodegenerative diseases. These effects of nicotine serve to emphasize the multifarious roles the drug, acting through multiple nicotinic acetylcholine receptor subtypes. The findings also remind us of the complexity of signaling mechanisms and stress the risks of unintended consequences of drugs designed to combat nicotine addiction.

Smoking, perhaps, is the number one preventable cause of serious illnesses like heart disease, stroke and cancer. The fact that nicotine is an addictive drug of abuse is undisputed. At the same time, the drug is thought to be neuroprotective in cases of neurodegenerative diseases like Alzheimer’s disease (AD) and Parkinson’s disease (PD), as well as in psychiatric disorders like depression and schizophrenia. How the drug mediates its effects is still largely unknown. Here we review what is known about nicotine’s actions on the brain and mechanisms that might contribute to its effects. Using two brain structures, the hippocampus and the Ventral Tegmental area (VTA), known to be involved in some or all effects of nicotine, as examples, we will sequentially address the following issues—a) properties and functional distribution of nicotinic acetylcholine receptors (nAChRs); b) signaling by nAChRs, c) what such mechanisms might inform us regarding nicotine addiction and the role of nAChRs in brain disorders and d) therapeutic approaches to combat these illnesses.

## Neuronal Nicotinic Receptors: Subtypes and Distribution

It is well accepted that the actions of nicotine are mediated by its ability to activate neuronal nicotinic receptors (nAChRs). The mammalian nAChR family consists of a number of subunits arising from a total of 11 gene products (α2–α7, α9 α10, β2–β4; Nicke et al. 2004), the α subunits being putative agonist binding subunits, based on homology with the muscle nicotinic receptor (α1). These receptors are arranged as pentamers with the five subunits forming a wall surrounding a central cationic ion channel. Activation of the receptors opens the central pore allowing cations to flux through, depolarizing neurons to their firing threshold.

### Distribution of nAChRs in the VTA and hippocampus

In the mammalian brain there are two predominant nAChR subtypes. The first is a homomeric receptor consisting of five α7 subunits (α7-nAChRs) and the second is a heteromer consisting of a combination of α4 and β2 gene products, with or without other subunits (α4β2*-nAChRs; the * reflects the potential heterogeneity in subunits. mRNA for almost all the common brain nAChR subtypes are found in VTA neurons (Charpantier et al. 1998; Champtiaux et al. 2002; Han et al. 2000). There are differences in abundance of message for various nAChR subunits between the hippocampus and the VTA. The mammalian hippocampus appears to contain mainly the α7 mRNAs while the VTA expresses a number of nAChR subunits to varying degrees. In the VTA, there are differences in subunit mRNA abundance between dopaminergic and non-dopaminergic neurons, with the latter mainly expressing α7 mRNAs (Table 1).

The two classes of nAChRs are identified by differences in their pharmacology (Table 2). α7-nAChRs have a relatively lower (μM) affinity for nicotine, compared to the heteromeric subtype, while they have a high affinity for the snake venom toxin alpha-bungarotoxin (αBTX). Thus, the distribution of the two receptor subtypes track with high affinity nicotine and αBTX binding (Seguela et al. 1993; Breese et al. 1997; Sorenson and Chiappinelli, 1992). As expected from mRNA distributions, nAChRs are widely distributed in the hippocampus and along the mesolimbic dopaminergic pathway. All dopaminergic neurons at the VTA appear to express a number of pharmacologically identified nAChRs, though only half of these neurons seem to possess the α7 subclass (Klink et al. 2001). Local GABAergic interneurons possess α4β2*-nAChRs (Klink et al. 2001). In the hippocampus, however, α7-nAChRs predominate in the stratum radiatum interneurons (Klein and Yakel, 2005) while heteromeric subtypes might exist in interneurons from other regions, e.g. stratum oriens (Khiroug et al. 2004). Using radiolabeled methyllycaconitine (MLA) an antagonist selective for the α7-nAChRs (Ward et al. 1990) and nicotine binding studies to discriminate between α7-nAChRs and α4β2*-nAChRs, it was shown that the hippocampus contained approximately two-fold greater α7-nAChR sites than the VTA but about 4-fold less of the α4β2*-nAChR sites (Mugnaini et al. 2002).

### Subcellular distribution

In neurons, functional nAChRs are not only widely distributed across different neuron types but are also differentially localized within a neuron—on the soma, dendrites or synaptic terminals. This diverse distribution of the receptors presents a challenge to the functional interpretation of receptor activation. It suggests a modulation of plasticity and activity at a network level more than linear modulations of individual synaptic pathways. In this section, we summarize evidence for functional localization of nAChRs.

#### Presynaptic terminals

nAChRs are present at presynaptic terminals of other transmitter systems where they modulate transmitter release. The key subtype mediating these effects is the α7-nAChR while other subtypes play a role as well. Together these receptors at presynaptic locations influence the release of glutamate (Gray et al. 1996; Sharma et al. 2008; Sharma and Vijayaraghavan, 2003), GABA (Lena and Changeux, 1997), norepinephrine (Clarke and Reuben, 1996), and dopamine (Grady et al. 2007), among others. Modulation of transmitter release at various nerve terminals could potentially be a mechanism for nicotine-mediated alterations in local synaptic plasticity. Small, spontaneous release events, increased by nicotine, can modulate local protein synthesis and affect synaptic efficacy (Sutton et al. 2004). At the mossy fiber terminals of the rat hippocampus, nicotine mediates an unusual form of plasticity. The drug causes a burst of glutamate release events in the absence of incoming action potentials, mediated by α7-nAChRs (Sharma and Vijayaraghavan, 2003). This burst consists of both an increase in glutamate release frequencies as well as a CaMKII-mediated concerted release of multiple quanta at these terminals (Sharma et al. 2008). Activation of α7-nAChRs induces a long-lasting slow calcium transient at these terminals resulting, at least partially, from the release of calcium from internals endoplasmic reticulum stores (Sharma et al. 2008). Most surprisingly, this burst of transmitter release is sufficient to drive the postsynaptic neuron above its firing threshold, in what is the first instance of a presynaptic action potential-independent transmission in the CNS (Sharma et al. 2008; Sharma and Vijayaraghavan, 2003). The relevance for this signaling mechanism for nicotine actions in vivo comes from the observation that at physiological temperatures, levels of nicotine found in the serum of smokers can mediate these effects (Sharma et al. 2008). The findings suggests that, at this synapse, nicotine ‘hijacks’ normal signaling pathways, altering synaptic strength in a manner independent of the physiological context but dependent on the presence of nicotine. Such modulation might have a very significant impact nicotine-mediated addictive processes and would be consistent with the more homeostatic view of addiction discussed below.

#### GABA interneurons

A common site for functional nAChRs appears to be at the soma and dendrites of GABAergic interneurons. In the VTA, local GABAergic neurons, as well as the feedback GABAergic input from the nucleus accumbens, contain α4β2*-nAChRs (Fagen et al. 2003; Mansvelder et al. 2003). These receptors, upon activation of cholinergic inputs might regulate the firing of the VTA output neurons controlling the patterning and timing of responses.

In the hippocampus, interneurons of the stratum radiatum, and other regions, contain α7-nAChRs. The receptors appear to be localized both at somatic and dendritic sites (Fayuk and Yakel, 2007; Klein and Yakel, 2006; Klein and Yakel, 2005) as well as at interneuron terminals where they modulate GABA release (Alkondon et al. 1999). However, α7-nAChRs are also present on secondary GABAergic interneurons and can thus mediate both inhibition as well as disinhibition of the output pyramidal neurons (Ji and Dani, 2000).

#### Principal neurons

There is evidence for the existence of functional nAChRs on dopaminergic output neurons at the VTA (Mansvelder et al. 2003). Putative α4β2*-nAChRs at somatodendritic sites might provide excitatory inputs for these neurons while α7-nAChRs at nerve terminals would enhance dopamine release at the nucleus accumbens, the target area. Coupled with the modulation of interneurons by nAChRs, these results suggest a complex regulation of the reward pathway by nAChRs.

In the hippocampus, evidence for the existence of nAChRs on the pyramidal neurons, the principal excitatory output neurons of the area, is more equivocal. While small nicotinic currents have been demonstrated from these neurons (Ji et al. 2001), other studies have failed to discern these signals (Khiroug et al. 2003).

#### Astrocytes

Studies over the last decade have demonstrated that astrocytes have more active role to play in modulation of synaptic activity than previously imagined. These cells have a form of excitability, not mediated by fast action potentials, but by slowly propagating calcium signals (Araque et al. 1999). In addition, accumulated evidence suggests an ability of these cells to communicate back to neurons by release of multiple vesicular and non-vesicular signals (Haydon, 2001).

Our studies showed that hippocampal astrocytes have functional α7-nAChRs that induce large calcium transients in these cells (Sharma and Vijayaraghavan, 2001). These signals result from a complex cascade of amplification upon receptor activation, discussed below. Presence of nAChRs on astrocytes *in situ* has been demonstrated as well (Gahring et al. 2005; Gahring et al. 2004a; Gahring et al. 2004b), suggesting another site for nicotinic modulation in the brain. As astrocytes are thought to be involved in controlling the local excitation of neuronal synapses, nicotinic control of these cells might contribute to the overall effects of the drug on synaptic function and plasticity.

**Figure 1:**
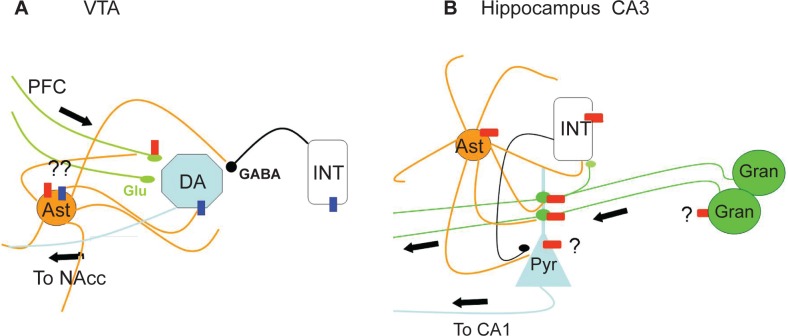
Intra-circuit distribution of functional nAChRs. **A)** α7-nAChRs (Red Boxes) and α4β2-nAChRs (Blue Boxes) distribution in the VTA. The homomeric receptors are predominant on the glutamatergic terminals of the inputs from the prefrontal cortex (PFC; Green lines). The heteromeric receptor is present on the GABAergic interneurons (INT) and on the Principal dopaminergic neurons (DA) which send their outputs to the nucleur accumbens (NAcc). The presence of functional nAChRs on astrocytes (Ast) from the VTA has yet to be demonstrated (denoted by **?**). Based on data from other systems, however, this is a distinct possibility. **B)** Distribution in the CA3 region of the hippocampus. The main functional evidence available is for the α7-nAChRs (Red Boxes). The homomeric receptor is on the GABAergic interneurons (INT) and at the mossy fiber boutons (Green circles) that are *en passant* terminals made on to the dendrites of the CA3 pyramidal cell (Pyr) and originating from the granule cells (Gran) of the dentate gyrus. Hippocampal astrocytes (Ast) possess functional α7-nAChRs. The presence of functional nAChRs on granule cells and the pyramidal cells are controversial (indicated by **?**). The nAChR modulated output is the axons of the CA3 pyramidal cells that innervate the Principal cells in the CA1 region. The figure illustrates the potentially complex modulation of network output by nAChRs.

In summary, functional nAChRs are widespread in their distribution among various cell types in the brain, controlling all aspects of signaling between neurons. This potentially complex modulation of network activity by nAChRs is illustrated in Figure 1. These findings suggest caution in linear interpretations of the drug effects in order to arrive at mechanistic correlates of behavioral changes induced by the drug.

## nAChR Signaling Mechanisms

The widespread incidence of nAChR-mediated rapid chemical transmission has yet to be demonstrated in the brain. In the stratum radiatum interneurons of the mammalian hippocampus, α7-nAChR synaptic currents were reported after the blockade of other major transmitter systems with antagonists (Frazier et al. 1998) while these were not observed in another study (McQuiston and Madison, 1999). Similarly, a fraction of synaptic currents recorded from CA1 pyramidal cells were consistent with nAChR responses (Hefft et al. 1999). However, a number of caveats plague these studies, the chief being the lack of information on the relationships between nAChRs, acetylcholine esterases (AChEs), and cholinergic innervation in an acute slice preparation, as well as an over reliance on pharmacological agents.

Both the unusual locations of these receptors, as well as the observations that very few postsynaptic specializations have been observed at ACh release sites (Contant et al. 1996), suggest a nontraditional role for nAChR activation, probably by transmitter diffusion.

Such an idea runs into the problem of having to account for receptor desensitization. All ligand-gated ion channels desensitize, some faster than others. Among nAChRs, the α7-nAChR subtype shows rapid desensitization with nicotine with a time constant of a few milliseconds, while the slowly desensitizing receptor types do so in the order of a few seconds (Zhang et al. 1994). It has been argued that at low doses of nicotine might desensitize the α4β2*-nAChRs but not the α7-nAChRs due to the fact that the latter has lower affinity for nicotine (Fenster et al. 1997; Mansvelder et al. 2003). While one cannot rule out the role of desensitization in mediating the behavioral effects of nicotine, the direct implication of this channel property might be difficult to demonstrate. First, channel kinetics and biophysical states of these receptors are not completely understood. Second, the assumption underlying these arguments, that nicotine concentrations seen in the serum of smokers are the same as that encountered by the receptors at synaptic and extrasynaptic sites, is not necessarily correct. In fact, nicotine might be present at higher concentrations in the brain and might have slower clearance rates (Ghosheh et al. 2001). Third, effects attributed to receptor desensitization might be effects downstream to receptor activation, e.g. presynaptic alterations in glutamate release probabilities in areas where nAChRs modulate its release. Lastly, the relationship between time courses of desensitization and inactivation (which are often used synonymously in nAChR literature) and nicotine-mediated changes in behavior are not intuitively obvious. The diffuse distribution of nAChRs makes it difficult to predict what the consequences of either activation or desensitization might be for network excitability and plasticity. In physiological context, it is also not obvious how channel desensitization would affect signaling based on ACh diffusion.

A unique feature of nAChRs, especially the α7-nAChR subtype, is that they have a high relative permeability for calcium and can also effectively raise intracellular free calcium concentration ([Ca]_i_). This ability of the receptors arises from being coupled to downstream calcium amplification mechanisms. Studies indicate that calcium flux through α7-nAChRs can be dramatically amplified by downstream release of calcium from ER stores via CICR (Sharma et al. 2008; Sharma and Vijayaraghavan, 2003; Vijayaraghavan et al. 1992). This ability of α7-nAChR to efficiently raise intracellular calcium levels plays a dominant role in the physiological function of the receptors and makes them effective mediators of downstream calcium signaling cascades (McKay et al. 2007; Dajas-Bailador and Wonnacott, 2004). The α4β2-nAChRs, on the other hand may play a more traditional role for ligand-gated ion channels which is to provide the initial depolarization for neuronal firing. At the same time, by activating voltage-gated calcium channels (VGCCs), these receptors would also play important roles in nAChR-mediated calcium signaling.

The time course of calcium signals generated by nAChRs as well as consequent physiological responses is also not consistent with receptor desensitization. In response to nicotine application, α7-nAChRs on mossy fiber terminals in the hippocampus show slowly rising calcium transients with decay times dependent on the duration of agonist application in the order of many seconds (Sharma et al. 2008). While most of the calcium signals observed in response to α7-nAChR activation comes from amplification via CICR, it still does not explain agonist exposure time-dependent signals, over periods up to 200s generated by a receptor that desensitizes in milliseconds (Sharma et al. 2008; Sharma and Vijayaraghavan, 2003). These results, coupled with many studies on the downstream effects of α7-nAChRs (e.g. Berger et al. 1998; Dajas-Bailador et al. 2000) suggest the possibility that a small, slow desensitizing component might be the relevant α7-nAChR signal mediating extrasynaptic and calcium-dependent effects of the receptor. If true, this would imply that measuring calcium signals might be a more sensitive assay for functional α7-nAChRs than whole cell current measurements. The idea remains to be tested.

Considering nAChRs primarily as modulators of calcium signaling rather than primary mediators of synaptic transmission makes the idea of volume transmission more feasible as they might not require fast and efficient delivery of ACh. Evidence for such a mechanism must, however, come from further studies on the nature of cholinergic signaling. For example, determining the extent of agonist diffusion requires adequate knowledge of the relative distribution of nAChRs, transmitter release sites, and local AChE concentrations. It is tempting to speculate that, unlike the neuromuscular junction, the AChE forms a pocket around release sites providing a reasonable distance for free diffusion of ACh enabling it to act on nAChRs within the pocket. This idea needs to be tested.

## Nicotinic Receptors in Disease States

### Autosomal dominant frontal lobe epilepsy (ADNFLE)

Some information on what role these receptors might play in CNS physiology comes from examining phenotypes of naturally occurring mutations in nAChRs genes. ADNFLE has been shown to be linked to nAChRs (Marini and Guerrini, 2007) which includes the α4 gene mutations (Phillips et al. 2000) as well as mutations in the β2 gene (Bertrand et al. 2005; Phillips et al. 2001). *In vitro* studies, using receptors reconstituted in Xenopus oocytes, have shown that relevant mutations to the α4 subunit results in a receptor response that shows increased desensitization and altered calcium permeability (Bertrand et al. 1998). In mice harboring the mutation, exposure to nicotine elicits a behavior termed as the dystonic arousal complex, a collection of symptoms akin to those seen in ADNFLE (Teper et al. 2007). Similarly, ADNFLE mouse models show an increased sensitivity for the seizure generating effects of nicotine (Klaassen et al. 2006). While these studies cannot be over generalized as nAChR mutations might account for only a subpopulation of ADNFLE sufferers, the results nonetheless demonstrate a correlation between altered nAChR activity and changes in neuronal excitability. Interestingly, many pathological nAChR mutations can be localized to regions predicted to affect ligand-induced channel gating from structural models (Taly et al. 2006).

### α7-nAChRs in schizophrenia

Schizophrenic patients show a much greater incidence of smoking than the general population (e.g. see Gopalaswamy and Morgan, 1986). These findings led to the examination of nAChRs in schizophrenia. (Bickford et al. 1993).

Auditory gating is measured as changes in a specific peak in EEG recordings. This response, known as the P50 auditory evoked response, is seen about 40–80 ms after the presentation of the auditory stimulus. In normal population presentation of two stimuli closely spaced in time (~500 ms) results in the attenuation of the P50 response to the second stimulus. This relative suppression of the P50 response is an indicator of sensory gating. There is much less suppression of the P50 evoked response in schizophrenics (Cullum et al. 1993), leading to the idea that defects in this process contribute to schizophrenic symptoms. Consistent with epidemiological data, smoking restores, to a large extent, the P50 ratios in schizophrenics (Adler et al. 1993) implying a role for nAChRs in this process. A number of studies indicate that P50 deficits show significant correlation with the level of α7-nAChRs in the brain (Adler et al. 1998). Further, infusion of α7-nAChR antagonists decrease P50 ratios (i.e. response to the second tone not suppressed) while agonists increase them (Simosky et al. 2003). This effect is mimicked by the atypical antipsychotic, clozapine, in a manner consistent with its effects being via α7-nAChRs (Simosky et al. 2003). Linkage analyses showed that the P50 changes were mapped to the chromosomal locus 15q13–q14 (Leonard et al. 2000). The α7 gene lies within this locus thus providing good correlation between α7-nAChRs, P50 deficits and schizophrenia (Freedman et al. 1997). These results also suggest that therapeutic interventions based on modulating α7-nAChR function might be useful in the treatment of certain schizophrenic symptoms as well.

### Nicotine and Alzheimer’s disease

A key finding over the years has been the role of α7-nAChRs in neuronal survival (Mechawar et al. 2004; Berger et al. 1998; Roy et al. 1998; Pugh and Margiotta, 2000; Dajas-Bailador et al. 2000) increasing the plausibility of a role for these receptors in neurodegenerative diseases.

Nicotine has been shown to increase memory and attention in normal humans (Warburton, 1992). In AD the drug has been shown to improve memory deficits. This idea is supported by epidemiological data suggesting that incidence of AD among smokers is significantly less than in non-smokers (Perry et al. 1999). The cholinergic hypothesis for AD has been prevalent for a long time based on the finding that loss of basal forebrain cholinergic neurons is one of the early symptoms of AD. This led to the use of acetylcholine esterase (AChE) inhibitors for treatment of the disease (Bartus et al. 1982). The results from this line of therapy have been disappointing, thus undermining the idea as a whole. Upon reflection, however, these findings are not contradictory to the cholinergic hypothesis. The efficacy of the AChE inhibitors, whose function is to increase the lifetime of the transmitter in the extracellular space, depends on the presence of cholinergic projections. If these are the earliest neurons to die, as suggested, the loss of projections to would render inhibition of AChE ineffective. More recent data suggests that nAChR agonists and antagonists might be a better, more effective, therapeutic approach to the disease. Some attempts at drug development based on this idea have been made. This is summarized in the next section.

A feature of α7-nAChRs is their modulation by the beta amyloid 1–42 peptide (Aβ), the key component of plaques found in the brain of AD patients. Aβ binds to the receptor with picomolar affinity (Wang et al. 2000) and it has been shown that the peptide, by activating α7-nAChRs, can modulate the MAPK pathway and CREB activation (Dineley et al. 2001). At the same time the receptor is down-regulated in AD brains (Oddo and LaFerla, 2006). In transgenic mouse models of AD, nicotine via the MAPK pathway increases the hyperphosphorylation of the *tau* protein, the cause of neurofibrillary tangles, which is a part of the AD pathology as well (Oddo and LaFerla, 2006; Oddo et al. 2005). Thus, the exact mechanisms which mediate the enhanced cognitive functions seen upon nicotine treatment remains unclear.

### Parkinson’s disease

Nicotine has been shown to be protective against PD as well (for a recent review see Singh et al. 2007). In PD, there is a specific loss of the dopaminergic neurons of substantia nigra, which provide inhibitory control to the neurons of the striatum. Once again the role of nAChRs is likely to be complex, involving differential modulation of a number of pathways. In mouse models of PD, where selective lesions of dopaminergic neurons were made by injection of 6-hydroxy dopamine (6-OHDA) selectively into the striatum or the substantia nigra, the levels of a number of nAChRs showed dramatic decline though the α7-nAChR levels remained unchanged (Jellinger, 2002). In patients with PD there is a selective increase in α7-nAChRs while levels of heteromeric nAChRs decline (Bordia et al. 2007; Janhunen and Ahtee, 2007). α7-nAChRs have been shown to trigger an anti-inflammatory pathway in brain microglia (Shytle et al. 2004). The activation of the receptor can suppress the inhibition of pro-inflammatory transcription factors NFkappaB and c-myc (Liu et al. 2007).

In neurons, dopamine is oxidized by monoamine oxidases (MAOs). One class of MAO; MAO-B, oxidizes dopamine and various primary and tertiary amines to their corresponding aldehyde and free amines, resulting in the release of hydrogen peroxide a source of free radicals. The oxidation of dopamine to dihydroxy phenyl acetic acid, via a series of reactions, generates a number of reactive oxygen species (ROS). As the brain has a more limited capacity to clear ROS than other tissues, these species can trigger a cytotoxic cycle, wherein in the presence of solvated Fe(II) and H_2_O_2_, the toxic 6-OHDA is formed (Fenton Reaction). The 6-OHDA, in turn, is able to mobilize more Fe(II) from stored forms of iron in proteins, thus propagating neurotoxicity and neuronal death. Nicotine has been shown to be neuroprotective by blocking MAO activity, thus acting as a protective antioxidant (Linert et al. 1999). This is discussed further under the section on clinical options (see below).

Thus there are both epidemiological, as well as potential mechanistic bases, for the protective role of nAChRs in neurodegenerative diseases and it is likely that α7-nAChRs play an important role.

## Nicotinic Receptors and Addiction

### Nicotine and smoking

The peak concentrations of nicotine reached in the bloodstream upon smoking a cigarette is ~0.5 μM–1 μM (Benowitz and Henningfield, 1994; Henningfield et al. 1993). However, nicotine appears to be concentrated in different compartments. In the brain, the concentrations of nicotine might be as much as five-fold that of the serum (Ghosheh et al. 2001). As the substance is hydrophobic and can also enter cells, it is almost impossible to determine concentrations at synapses. This becomes an interpretational problem, as discussed below.

### Potential mechanisms underlying nicotine addiction

The fact that nicotine is an addictive drug is no longer in dispute, if it ever was. On the other hand, the mechanistic basis of this process is still far from clear.

A large body of literature has focused on the role of the mesolimbic dopaminergic circuit in addiction, linking addiction to reward seeking behavior. In 1958, James Olds published a series of papers identifying this system as the ‘pleasure centers’ of the brain. These studies demonstrated that electrical stimulation of these areas resulted in a powerful positive drive in animals that proved stronger than other innate drives like hunger and self-preservation. Further, placing electrodes in this region resulted in a strong self-stimulation by the animals (OLDS, 1958b; OLDS, 1958a; OLDS, and OLDS, 1958; OLDS, 1958c). The link between these studies an addiction, however, came a couple of decades later, with studies demonstrating that delivering drugs of abuse, like cocaine, at the reward centers identified by Olds resulted in the same behavior resulting in an equally strong drive to self-administer these agents (Goeders and Smith, 1983). The findings resulted in a paradigm shift in our view of addiction changing from a negative drive, i.e. to avoid withdrawal symptoms, to a positive connotation to the drugs themselves.

In the subsequent years, a vast literature was generated showing the action of various drugs on the mesolimbic reward system (McClung and Nestler, 2008; Koob and Nestler, 1997; Nestler, 1994a; Nestler, 1994b; Nestler et al. 1993; Nestler, 1992).

The mesolimbic reward system originates as a bundle of dopaminergic fibers at the ventral tegmental area (VTA) and fans out to a number of limbic areas including the nucleus accumbens (NAcc) and the prefrontal cortex. Dopaminergic neurons of the VTA are under the control of both GABAergic interneurons as well as incoming glutamatergic inputs from the prefrontal cortex. These glutamatergic inputs into the VTA provide the main excitatory control for dopamine release to further downstream areas of the pathway (Sesack and Pickel, 1992; Taber et al. 1995; Taber and Fibiger, 1995). Some of these inputs also terminate at the nucleus accumbens potentially providing a positive feed forward mechanism at the reward pathways (Sesack and Pickel, 1992).

There appear to be two pathways by which drugs of abuse modulate the mesolimbic dopaminergic system: the first is the direct alteration of signaling at the VTA and the second is the modulation of dopamine release at terminals in the NAcc. Nicotine appears to modulate both the excitatory as well as inhibitory inputs on the dopaminergic neurons of the VTA.

### Drug Addiction: A homeostatic view

The prevailing wisdom is that addiction is a learnt behavior, which implies that like other forms of learning and memory, the consequence of nicotine exposure would be a net increase in long-term synaptic strength. It is being recognized that widespread distribution of receptors for drugs of abuse implies that their action is not restricted to the mesolimbic reward pathway. This leads to a paradigm shift in our way of thinking about addiction and other actions of these drugs. nAChRs are present in a number of cortical, midbrain, and hindbrain loci.

An attractive concept put forth a few years ago by Koob and colleagues. The idea is that drug addiction is a means of self-medication for maintaining hedonic homeostasis in the brain- an acceptable balance of positive and negative affective states. According to this theory, drug addiction is a complex phenomenon involving a lot more than just activation of reward pathways. It also links the addictive process to emotional dissonance explaining the greater prevalence of drug abuse among people with mood disorders. This, more inclusive, viewpoint most certainly involves a host of neurotransmitter systems in a number of areas of the brain.

The term ‘allostasis’ is used to refer to this condition where the brain must vary all its parameters to match them to perceived environmental demands, in order to maintain stability. This also implies that when stability is achieved, it includes the additional external variable, the drug, and withdrawal of the drug would consequently lead to instability. One consequence of this instability in addicts would be a negative hedonic balance, leading to craving for the drug, attentional bias in the form of preoccupation and anticipation, all of which lead to continued drug use or relapse (Koob and Le Moal, 1997; Koob, 1996). Changes in synaptic strength induced by previous exposure to addictive drugs would now lead to spiraling distress as the brain attempts to reach a new homeostasis.

There are a number of attractive facets to this idea. First, it implies that a vast number of experiences, drug related or not, can be addictive, a conclusion intuitively attractive. Second, it takes into account the non-linearity of signal processing in the brain, raising the idea that it is possible to arrive at a common endpoint from a number of different circumstances. Such a mindset would also be valid for other psychiatric disorders. Third, it allows for a common perspective for the action of a number of addictive drugs that have a wide range of targets and end results and also provides an easier explanation for the small degree of co-morbidity among drugs of abuse. Fourth, as the brain will go into a long lasting dynamic instability until it finds a new, acceptable, homeostatic state, it also predicts the possibility of relapse after considerable periods of abstinence. Lastly, it recognizes the importance of the individual in the addiction process, implying that the strength and nature of the drugs abused would depend on the initial and the end hedonic state of the individual. It drives addiction research from searching for common brain pathways to more individually tailored hypotheses, implying that it might be futile to look for a one size fits all treatment approaches. This is definitely borne out in clinical studies with various treatment options to tobacco addiction.

The wide distribution of nAChRs is certainly consistent with this view. Our recent findings that activation of α7-nAChRs in the hippocampus leads to synaptic transmission independent of information coming down the presynaptic axon (Sharma et al. 2008; Sharma and Vijayaraghavan, 2003). This is consistent with the idea of allostasis in that nicotine usurps normal signaling pathways resulting in strengthening of synapses in non-physiological contexts. Such an altered homeostatic state is now dependent on the presence of the drug, the withdrawal of which would result in instability and distress.

## Current Treatment Options

A number of treatment options are currently available or in the development process to target nicotine addiction. In this section, we will briefly summarize some of the more promising approaches that can augment, or substitute for, current nicotine replacement therapies (Table 3).

### Nicotine Vaccines

A recent approach to tackling nicotine addiction is to raise antibodies against the drug to prevent its access to the brain. A number of companies are developing such vaccines (Novartis, Sanofi-Aventis, Nabi Biopharmaceuticals, Xenova Group). The idea is to link nicotine, as a hapten, to carrier proteins like the bacteriophage Qβ coat protein (Cornuz et al. 2008), in order to make it an effective immunogen.

While initial studies have shown this approach to result in a statistically significant increase in the rates of abstinence among smokers, the long-term efficacy of this approach remains to be determined. While no serious side effects of this treatment have been noted in humans, a number of issues need to be resolved and await a longer-term examination of this approach (LeSage et al. 2006b; LeSage et al. 2006a). A risk, is that, as the vaccine might only sequester a fraction of the serum nicotine, it might induce patients to smoke more. While this concern has not been borne out from limited studies, it still needs to be resolved. A second issue is one of withdrawal. As would be expected, in the presence of nicotine antibodies, the drug does not alleviate its own withdrawal symptoms. An interpretation of this outcome would be that nicotine use would no longer be rewarding to ameliorate withdrawal symptoms and therefore be less reinforcing. However, a converse argument, based on the viewpoint of addiction outlined above, would be the following—if the driving need for hedonic homeostasis persists, then there would be an increased risk that as nicotine fails to serve the need, other drugs might be sought after to fill the need. The danger that one would merely be swapping drugs needs to be appreciated.

### Altering nicotine metabolism

Once it enters the bloodstream, nicotine is rapidly metabolized in the liver. The immediate reaction step is the conversion of nicotine to its Δ^1′(5′)^—iminium form. This conversion is catalyzed by the Cytochrome P450 enzyme CYP2A6. The iminium form is rapidly oxidized to a major nicotine metabolite cotinine. CYP2A6 is the key enzyme in this pathway, also catalyzing the conversion of cotinine to t-3^′^-Hydroxycotinine (Hukkanen et al. 2005; Dempsey et al. 2004). CYP2A6 is a highly polymorphic gene, with at least 23 numbered variants differing in their expression, stability and activity. In a manner analogous to Alcohol dehydrogenase, these variants determine the clearance rate of nicotine and thus affect smoking behaviors. (Nakajima and Yokoi, 2005; Yamanaka et al. 2005). In a few studies methoxsalen, a CYP2A6 inhibitor reduced subjects’ desire to smoke thus increasing latency to the lighting of the next cigarette (Siu and Tyndale, 2008; Sellers et al. 2003a; Sellers et al. 2003b).

Further studies are under way evaluating the efficacy of CYP inhibitors coupled with other approaches like nicotine replacement therapy.

### nAChR agonists and antagonists

The use of nAChR agonists and antagonists has been the area targeted by the bulk of pharmaceutical research over the last few years. A number of candidate drugs have been developed that are either in the market or at various testing stages.

Varenicline, a drug based on the alkaloid cytisine, is a partial agonist at the α4β2*-nAChRs and a full agonist at α7-nAChRs (Mihalak et al. 2006) and has been approved by the FDA for smoking cessation treatments. While the relative action of this drug on the two nAChR subtypes vis a vis its efficacy in smoking cessation is not clear, it is assumed that it alters the mesolimbic reward system by modulating dopamine release (Stack, 2007).

The marine worm toxin, anabasine has been the base compound for other drugs aimed at targeting nAChR effects. Two benzylidine derivatives of this compound GTS-21 and 4OH GTS-21, which have specific agonistic properties for the α7-nAChRs (Uteshev et al. 2003), are under Phase II trials for the treatment of Schizophrenia (Freedman et al. 2008). An anti-emetic drug, Tropisetron, has also shown potential for the treatment of auditory gating deficits in Schizophrenia (Koike et al. 2005).

### Other approaches

There are other treatment options being considered to treat nicotine addiction that use other, potentially downstream targets of the drug (see George and O’Malley, 2004). These demonstrate significant efficacy, either by themselves or in combination with other treatments. The hedonic homeostasis view of drug abuse postulates that addiction is a form of mood disorder. Consistent with this viewpoint, a number of drugs like antidepressants have been found to be efficacious in combating nicotine addiction.

Smokers exhibit lower levels of MAO levels compared to non-smokers (Fowler et al. 1996; Fowler et al. 2003). As MAOs are key enzymes in modulating dopamine levels in the brain, it was postulated that maintaining low levels of the enzyme would be useful in combating nicotine addiction. The MAO inhibitor Moclobemide has been shown to help smoking cessation (Berlin et al. 1995b; Berlin et al. 1995a). Another MAO inhibitor Selegiline, has shown efficacy in smoking cessation (George et al. 2003), though a recent study shows the drug to also be a potent inactivator of CYP2A6 in humans (Siu and Tyndale, 2008).

Tricyclic antidepressants, like Nortriptyline, have been shown to reduce craving symptoms during early smoking abstinence periods (Prochazka et al. 1998; Hall et al. 1998). Similarly, the more atypical monoamine transporter blocker and antidepressant, Bupropion, has been marketed as aid for smoking cessation (Hays et al. 2001; Hurt et al. 1997). The μ-opiod receptor antagonist, naltrexone, has been shown to reduce nicotine craving when provided in conjunction with transdermal nicotine patches (Wong et al. 1999), by acting as an anxiolytic and antidepressant (Jarvekulg and Viru, 2002) and, possibly, by acting as an open channel blocker of the α7-nAChRs (Almeida et al. 2000).

Future attempts at using individually tailored combinations of these various approaches might lead to more effective therapeutic options to combat both smoking behaviors and diseases like AD and PD. This remains to be seen.

## Conclusion

The importance of combating nicotine addiction is unquestionable. A number of nAChR agonists and antagonists are candidates for the treatment of addiction and neurodegenerative disorders. However, the widespread distribution of nAChRs adds a degree of caution in this regard. It should be recognized that understanding the physiological context in which these receptors work and consequences of functional disruption must give us pause. While it would be impractical to halt drug development until physiology is known, it is also imperative that we pay very close attention to the possibility of unintended consequences of such drugs.

Another important paradigm shift would be the recognition of addiction as a non-linear process with multiple possibilities of homeostatic disruptions leading to a similar phenotype. This view would imply that any single drug would be effective only in a small subpopulation of addicts and investing resources for a universal treatment for nicotine addiction might be a futile exercise.

## Figures and Tables

**Table 1:** Comparison of nAChR subunit mRNA expression in hippocampus and VTA.

Region	α3	α4	α5	α7	β2	β4
Hippocampus	+	+	+	++	+	+
Dentate	+	+	+	++	+	+
Prefrontal	++	++		+++	+	
VTA DA neurons	++	++	++	++	++	++
VTA Non-DA neurons	+	+	+?	++	+	++

Relative distribution of common nicotinic subunit mRNAs in the hippocampus and VTA. (?) indicates conflict among reports. Data compiled from (Court JA et al. 1995; Breese et al. 1997; Rubboli et al. 1994; Ostermann et al. 1995; Lobron et al. 1995; Azam et al. 2003; Azam et al. 2002; Klink et al. 2001).

**Table 2:** Functional nAChR pharmacology.

Subtype	Agonist	EC_50_ (μM)	Antagonists	IC_50_ (μM)
α7-nAChRs	ACh	130^1^, 155^2^	αBTX	0.0016^5^
	Nicotine	49^2^, 27^3^	MLA	0.00025^6^
	Choline	1600^1^, 493^4^	Conotoxin ImI	0.022^7^
			Mecamylamine	15.6^8^
α4β2*-nAChRs	ACh	2.1^3^	DHβE	0.08^10^
	Nicotine	0.9^9^	Mecamylamine	0.77–2.3^8^

Data compiled from 1) Alkondon et al. 1997; 2) Gopalakrishnan et al. 1995; 3) Alkondon and Albuquerque, 1993; 4) Gonzalez-Rubio et al. 2006; 5) Zhang et al. 1994 6) Palma et al. 1996; 7) McIntosh et al. 1999; 8) Papke et al. 2008; 9) Alkondon and Albuquerque, 1995; 10) Buisson et al. 1996.

**Table 3:** Potential therapeutic compounds targeting nAChRs.

Drug	Target	Postulated effects	Potential use
Nicotine Vaccine	Serum Nicotine	Blocking drug entry into the brain	Smoking cessation
Varenicline	α4β2*- and α7-nAChrs	Affects mesolimbic dopamine levels	Smoking cessation
GTS-21 and 4OH GTS-21	α7-nAChRs	Improves auditory gating	Schizophrenia
Tropisetron	α7-nAChRs	Improves auditory gating	Schizophrenia
TC-1698	α7-nAChRs	Neuroprotection	AD and PD
Methoxsalen	CYP2A6 inhibitor	Alter nicotine clearance	Smoking cessation
Selegiline	CYP2A6 blocker, MAO inhibitor	Affects nicotine clearance, dopamine metabolism	Smoking cessation, PD
Moclobemide	MAO inhibitor	Alters Dopamine metabolism	Smoking cessation, Depression
Buproprion	Dopamine Transporter Blocker	Affects mesolimbic dopamine levels	Smoking cessation and Depression
Nortriptyline	NE/5HT transporter blocker	Increases brain NE and serotonin levels	Smoking cessation and Depression
Naltrexone	μ-opioid antagonist and α7-nAChR channel blocker	Anxiolytic and antidepressant	Smoking Cessation
